# 

**Published:** 1873-02

**Authors:** 


### Contents of February Number.

### ORIGINAL DEPARTMENT.

ART. I. Are the public competent to judge what system of medicine is most desirable, or what practice they should adopt. By T. D. Washburn, M. D., Hillsboro, Ill. . . . . . . . . 241
ART. II. Medical Society of the County of Albany. Semi-Monthly Meeting. Reported by F. C. Curtis, M. D., . . . . . . 250
ART. III. Epidemic Cerebro-Spinal Meningitis. By Chas. Forbes, M. D., Rochester, N. Y., . . . . . . . . . . 256

### MISCELLANEOUS.

Clinical Notes on the Electric Cautery in Uterine Surgery. By J. Byrne, M. D. 262
Medical Society of the State of New York. Sixty-Seventh Annual Meeting. Reported by F. C. Curtis, M. D., . . . . . . . . 271

### EDITORIAL DEPARTMENT.

Changing the Ethics of the Medical Profession, . . . . . . . 277
Commencement of Buffalo Medical College, . . . . . . . 277
State Medical Society, . . . . . . . . . . . . . 278

Books Reviewed:

Wholer's Outlines of Organic Chemistry, . . . . . 278
A Hand Book of Therapeutics. By Sidney Ringer, M. D., . 278
Surgical Diseases of Infants and Children. By M. P. Guersant, 279
Obstetric Aphorisms. By Joseph G. Swayne, M. D., . . 279

Books and Pamphlets Received, . . . . . . . . . . . 240

